# Temperature and Moisture Dependent Dielectric and Thermal Properties of Walnut Components Associated with Radio Frequency and Microwave Pasteurization

**DOI:** 10.3390/foods11070919

**Published:** 2022-03-23

**Authors:** Yuxiao Mao, Yujun Hao, Xiangyu Guan, Penghao Wang, Shaojin Wang

**Affiliations:** 1 College of Mechanical and Electronic Engineering, Northwest A & F University, Yangling 712100, China; maoyuxiao@nwafu.edu.cn (Y.M.); haoyujun@nwafu.edu.cn (Y.H.); xiangyuguan@nwafu.edu.cn (X.G.); wangpenghao@nwafu.edu.cn (P.W.); 2 Department of Biological Systems Engineering, Washington State University, Pullman, WA 99164-6120, USA

**Keywords:** dielectric properties, thermal properties, walnut components, pasteurization, radio frequency, microwave

## Abstract

To provide necessary information for further pasteurization experiments and computer simulations based on radio frequency (RF) and microwave (MW) energy, dielectric and thermal properties of walnut components were measured at frequencies between 10 and 3000 MHz, temperatures between 20 and 80 °C, and moisture contents of whole walnuts between 8.04% and 20.01% on a dry basis (d.b.). Results demonstrated that dielectric constants and loss factors of walnut kernels and shells decreased dramatically with raised frequency within the RF range from 10 to 300 MHz, but then reduced slightly within the MW range from 300 to 3000 MHz. Dielectric constant, loss factor, specific heat capacity, and thermal conductivity increased with raised temperature and moisture content. Dielectric loss factors of kernels were greater than those of shells, leading to a higher RF or MW heating rate. Penetration depth of electromagnetic waves in walnut components was found to be greater at lower frequencies, temperatures, and moisture contents. The established regression models with experimental results could predict both dielectric and thermal properties with large coefficients of determination (*R*^2^ > 0.966). Therefore, this study offered essential data and effective guidance in developing and optimizing RF and MW pasteurization techniques for walnuts using both experiments and mathematical simulations.

## 1. Introduction

Walnuts (*Juglans regia* L.) have experienced fast-growing harvest areas and annual yields due to their unique and favorite flavor and taste, and a rapid increase in the value of production. The largest producer of in-shell walnuts in 2022 was China, where over 30% (1.10 Mt) of the global production (3.32 Mt) was produced [1]. Fresh walnuts are susceptible to decay during storage due to microbial spoilage. Many bacterial pathogens, especially *Salmonella,* have been detected in walnuts [2,3]. In addition, aflatoxin contamination in walnuts during storage is the primary cause of food safety problems and mainly results from the infection of *Aspergillus flavus,* *A. niger*, and *Penicillium citrinum* [4]. For in-shell walnuts, the contamination of pathogenic bacteria is firstly on shells, and usually transfers to kernels during processing and storage [5]. To minimize the influence of potential spoilage on quality deterioration and reduce the population of bacterial pathogens and mold to a level qualified for consumption, pasteurization treatments for in-shell walnuts are crucial.

Some traditional methods have been investigated as pasteurization techniques for agricultural products. However, pasteurization by hot water at 76 °C for 3 min only achieves a reduction of about 1 log CFU/g of total plate count (TPC) on the surface of cantaloupe and is not suitable for dried products [6]. Steam provides a short pasteurizing duration of 25 s and a 5 log CFU/g reduction of *Salmonella* serotype Enteritidis [7], but the high processing temperature might lead to quality deterioration. Therefore, exploring alternative advanced technologies is desirable to develop an effective, practical, and economically viable method for pasteurizing in-shell walnuts.

Dielectric heating, including radio frequency (RF) and microwave (MW) treatments, raises temperatures of materials quickly and volumetrically since the heat generates inside products by dipole rotation and ionic migration as subjected to an alternating electromagnetic (EM) field [8]. Owing to the rapid heating, shortened processing duration, maintained product quality, and no chemical residues, the RF and MW treatments have been studied as novel pasteurization methods for nuts and other agricultural products, such as hazelnuts [9], green beans [10], walnuts [11], and paprika [12]. RF wave is more suitable for the treatment of bulk and thick materials because of the more uniform heating caused by the larger penetration depth as compared with MW processing [13]. By contrast, MW wave is commonly used for pasteurizing thin layer materials owing to the higher heating rates at larger frequencies [14]. Dielectric properties (DPs) of walnuts are essential data used to analyze the efficiency of MW and RF pasteurization processes. Mathematically, DPs are expressed in the form of relative complex permittivity (*ε*) as follows:

(1) $$\varepsilon =\varepsilon^{\prime }-j\varepsilon^{\prime \prime }$$

where *j* is the imaginary unit, $\sqrt{-1}$. The real part, *ε′*, is dielectric constant, representing the capacity of materials to store the electric energy. The imaginary part, *ε″*, is dielectric loss factor and refers to the ability of transforming electric energy into thermal energy. During RF or MW heating for agricultural products, thermal properties (TPs) containing specific heat capacity (*C_p_*, J/(kg·K)) and thermal conductivity (*k*, W/(m·K)) are also vital factors influencing the absorption and conduction of the thermal energy [15]. Specific heat capacity indicates the amount of heat required to raise the temperature of 1 kg of materials by 1 K without any phase or chemical changes, while thermal conductivity refers to the rate of heat transfer within materials. Nevertheless, both dielectric and thermal properties are mainly affected by some factors, such as temperature, moisture content, and frequency. Consequently, a comprehensive understanding of dielectric and thermal properties of walnuts is important for the effective design and improvement of pasteurization processes using RF or MW energy [16].

Several studies have investigated the dielectric and thermal properties of agricultural products as subjected to different ranges of frequency, moisture content (MC), and temperature for various purposes [17,18,19,20]. Specifically, Zhu et al. (2014) studied the dielectric properties of hazelnut kernels within a frequency band from 10 to 4500 MHz, a temperature range of 20–60 °C, and a MC range of 4.6–20.3% on a wet basis (w.b.) using an open-ended coaxial method [21]. The results showed that dielectric constant and loss factor of hazelnut kernels raised with increasing temperature, MC and falling frequency. Boldor et al. (2004) found that dielectric properties of both in-shell and shelled peanuts increased with increasing density and applied dielectric theory mixture equations to correlate these two parameters [22]. In terms of thermal properties, Huang et al. (2016) reported that both specific heat capacity and thermal conductivity of soybeans increased with raised temperature from 20 to 80 °C and MC from 4.64% to 7.86% (w.b.) [23]. Similarly, Perussello et al. (2014) chose Okara as an object, which is the residue of the soy beverage and tofu production and observed that thermal properties of Okara decreased with reduced moisture [24]. In the nut processing industry, shelling is a procedure after the pasteurization, so in-shell walnuts are common materials used for pasteurization treatments. Walnut kernels contain mainly fat and protein whereas lignocellulose in shells accounts for more than 80%, leading to a large difference between their dielectric properties [25,26]. In addition, to reduce the thermal resistance of bacteria, the MC of walnuts is commonly raised by adding distilled water before the pasteurization and removed by drying after the pasteurization. Therefore, dielectric properties of walnuts vary greatly as the moisture is evaporated continuously during the pasteurization and subsequent drying. However, to the authors’ knowledge, DPs of walnuts were only determined by Wang et al. (2003) at a single MC of 3% (w.b.) for kernels [27]. Furthermore, the information about dielectric and thermal properties of walnut components including kernels and shells is indispensable for further investigations in dielectric pasteurization treatments but unavailable at various MCs, temperatures, and frequencies.

Therefore, the objectives of this study were (1) to determine the dielectric properties of walnut kernels and shells within a frequency range of 10–3000 MHz at temperatures varying from 20 to 80 °C and four MCs of whole walnuts (8%, 12%, 16%, and 20% d.b.), (2) to measure the thermal properties of kernels and shells at a temperature range of 20–80 °C and the four MC levels of whole walnuts, (3) to provide empirical equations describing dielectric and thermal properties of walnut kernels and shells as affected by MC and temperature, and (4) to calculate the penetration depth of electromagnetic energy into kernels and shells of walnuts at the four representative frequencies (27, 40, 915, and 2450 MHz).

## 2. Materials and Methods

### 2.1. Materials and Sample Preparation

Dried and raw in-shell walnuts (*Juglans regia* L.) were purchased from a local farm market in Yangling, Shaanxi, China. The integrity of shells was adopted as the criterion of selecting walnuts for experiments. The selected walnuts with intact shells were then stored in polyethylene bags inside a refrigerator at 4 °C until further handling to avoid moisture loss and inhibit quality deterioration in ambient conditions [28]. For obtaining walnut kernels and shells with different levels of MC for further determining dielectric properties, four MC levels of 8%, 12%, 16%, and 20% (d.b.) were chosen as target MCs of whole walnuts based on the pasteurization treatment and a MC of 8%, which is qualified for long-term storage of walnuts in the ambient environment [11,29]. Distilled water with different pre-calculated quantities was added to the polyethylene bags with in-shell walnuts to adjust the MCs to the target ones. These in-shell walnuts were subsequently sealed in polyethylene bags at 4 °C for the complete absorption of water for 7 d when the bags were shaken twice a day to ensure the uniform distribution and complete absorption of the added water. Finally, four MCs of 8.04 ± 0.28%, 12.11 ± 0.08%, 15.91 ± 0.26%, and 20.01 ± 0.82% (d.b.) were obtained for in-shell walnuts, while the MCs of walnut components were adjusted to 4.21 ± 0.32%, 8.08 ± 0.06%, 12.08 ± 0.40%, and 16.23 ± 0.61% (d.b.) for kernels, and 12.51 ± 0.25%, 16.08 ± 0.14%, 20.07 ± 0.10%, and 23.86 ± 1.59% (d.b.) for shells, respectively.

### 2.2. Measurements of Moisture Content and True Density

The MC measurement was conducted following the AOAC Official Method 925.40 [30]. After separating shells of five stochastically chosen walnuts from the kernels, the two parts were separately ground by a blender (DE-300 g, 30–300 mesh, Zhejiang Hongjingtian Co., Ltd., Jinhua, China). Then, the powders of kernels and shells were placed in a vacuum oven (DZX-6020 B, Nanrong Lab equipment Inc., Shanghai, China) at a temperature of 105 °C and a pressure of 13.3 kPa and dried to a constant weight for about 5 h. To avoid the rapid absorption of moisture in the air by powders under the high temperature, a desiccator was applied to cool the samples to ambient temperature immediately after the drying. The MC of samples was expressed as the percentage of lost weight to the remaining one.

True density is a necessary parameter for preparation of walnut samples for dielectric property measurements. A liquid displacement method, of which the procedures were described in detail in Guo et al. (2008), was employed to determine the true density of walnut kernels and shells at different MCs [31]. To avoid being absorbed by samples, toluene (C7H8) was used as the displacement liquid because of the small surface tension. For each MC level, shells and kernels of five randomly chosen in-shell walnuts were separated and cut into small pieces. In each measurement, about 3 g samples were weighed and then placed into a 250 mL Lee’s pycnometer with toluene, and the volumes occupied by samples were recorded. The true density was calculated by dividing the weight by the volume. Each test for measuring densities of both walnut shells and kernels was conducted in triplicate at each MC.

### 2.3. Measurements of Dielectric Properties

An open-ended coaxial probe system, consisting of an impedance analyzer (E4991 B-300, Keysight Technologies Co., Ltd., Palo Alto, CA, USA), a matched calibration kit (E4991 B-010), a high-temperature coaxial cable, the coaxial probe with dielectric probe kit (85070 E-020), a cylindrical customized sample test cell, an oil circulated bath (SST-20, Guanya Constant Temperature Cooling Technology Co., Ltd., Wuxi, China), and an auxiliary computer, was adopted to measure the dielectric properties of walnut kernels and shells within a frequency range between 10 and 3000 MHz. The detailed structure of the system was described by Li et al. (2017) [32].

Before the dielectric property determination, two calibrations were carried out. For the first calibration, the impedance analyzer was switched on for at least 20 min to warm up, and then calibrated using open, short, and 50 Ω load successively. Subsequently, the open-ended coaxial probe was attached to the system and calibrated by air, short circuit, and 25 °C deionized water in the order of the second calibration. To ensure the tight contact between the probe and samples to eliminate air gaps, kernels and shells of five stochastically selected walnuts were milled into powders, and about 12 g samples were put into the sample test cell (21 mm diameter × 50 mm height) and compressed into cylinders prior to experiments. Temperatures from 20 to 80 °C with an interval of 10 °C were adopted based on the maximal range commonly used for pasteurizing in-shell walnuts [11]. The temperature of samples was monitored by a thermocouple (HH-25 TC, Type-T, OMEGA Engineering Inc., Stamford, CT, USA) and maintained at selected levels by the oil bath. Each test for measuring dielectric properties of both walnut kernels and shells was performed in triplicate at each temperature and MC.

### 2.4. Measurements of Thermal Properties

A thermal property analyzer (KD2 Pro, Decagon Inc., Pullman, WA, USA) combining with an SH-1 sensor was used for determining thermal properties involving thermal conductivity and specific heat capacity of walnut kernels and shells. For each measurement, samples with different MCs were placed into the test cell and pressed into cylinders following the same aforementioned procedure. Subsequently, the Type-T thermocouple and the SH-1 sensor were inserted into the center of samples. The same temperature range of 20–80 °C with an interval of 10 °C as that for measuring DPs was employed. After each predetermined temperature was reached by the oil bath, the thermal property analyzer was turned on for measuring thermal properties. Each test for measuring thermal properties of both walnut kernels and shells was also conducted in triplicate at each temperature and MC.

### 2.5. Determination of Penetration Depth

Penetration depth (*d_p_*, m) of MW and RF energy is defined as the distance travelled by the electromagnetic wave when the power declines to 1/e (e = 2.718) of the initial one at the entry surface of the material [33]. The penetration depth was determined by the following equation:

(2) $$d_{p}=\frac{c}{2\pi f\sqrt{2\varepsilon^{\prime }\left[\sqrt{1+{\left(\frac{\varepsilon^{\prime \prime }}{\varepsilon^{\prime }}\right)}^{2}}-1\right]}}$$

where *c* is the speed of light in free space (3 × 10^8^ m/s) and *f* is the frequency (MHz) of electromagnetic wave.

### 2.6. Statistical Analysis

The experimental results were expressed as mean ± standard deviations over all three replicates. A significance test was performed using the statistical analysis software SPSS 23.0 version (SPSS Inc., Chicago, IL, USA). Differences (*p* ≤ 0.05) between means were estimated by analysis of variance (ANOVA) and Tukey’s post hoc test.

## 3. Results and Discussion

### 3.1. True Density

Table 1 lists true densities of walnut kernels and shells with different MCs. True density of kernels decreased from 1.019 to 1.003 g/cm^3^ as the MC rose from 4.21% to 16.23% (d.b.). However, true density of shells increased from 0.988 to 1.067 g/cm^3^ with increasing MC from 12.51% to 23.86% (d.b.). Similar results are also reported in the research on hazelnuts [34] and Turkish walnuts [35], which might be caused by greater increase proportions of volumes than those of weights for kernels with added moisture. Since the variation of the true density in both walnut kernels and shells was negligible, the effect of density on the dielectric properties was neglected in this study. As a result, the true densities of walnut kernels and shells used for determining thermal and dielectric properties were defined as their means of 1.010 and 1.030 g/cm^3^, respectively.

### 3.2. Frequency-Dependent Dielectric Properties

**Figure 1:**
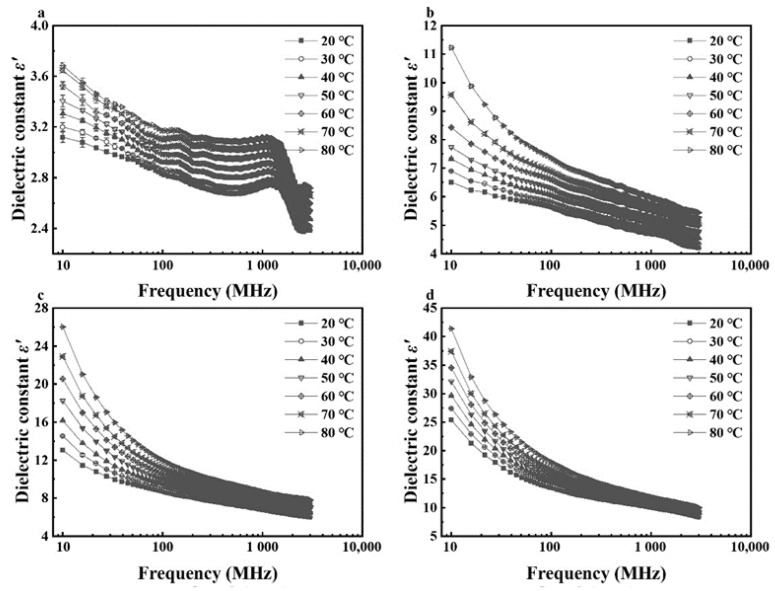
Frequency-dependent dielectric constant (*ε′*) of walnut kernels at 7 temperatures with moisture contents of 4.21% (**a**), 8.08% (**b**), 12.08% (**c**), and 16.23% (**d**) (d.b.).

**Figure 2:**
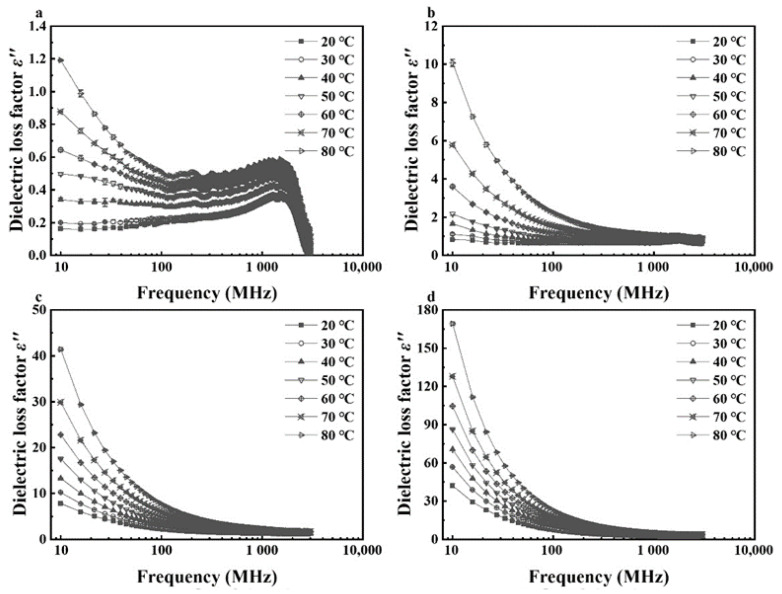
Frequency-dependent dielectric loss factor (*ε″*) of walnut kernels at 7 temperatures with moisture contents of 4.21% (**a**), 8.08% (**b**), 12.08% (**c**), and 16.23% (**d**) (d.b.).

**Figure 3:**
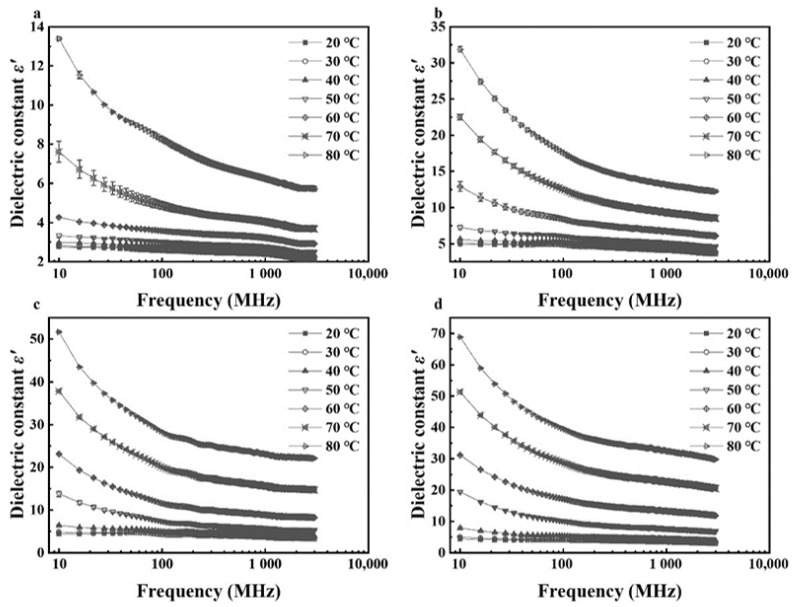
Frequency-dependent dielectric constant (*ε′*) of walnut shells at 7 temperatures with moisture contents of 12.51% (**a**), 16.08% (**b**), 20.07% (**c**), and 23.86% (**d**) (d.b.).

**Figure 4:**
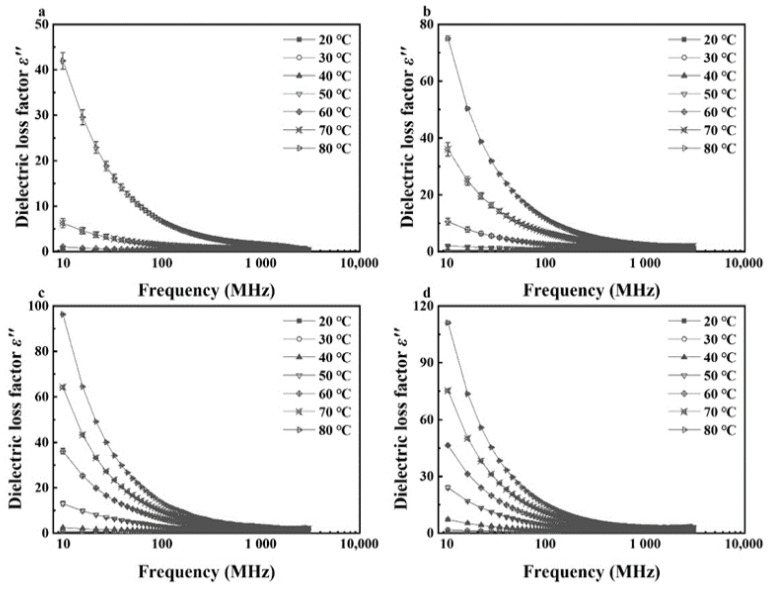
Frequency-dependent dielectric loss factor (*ε″*) of walnut shells at 7 temperatures with moisture contents of 12.51% (**a**), 16.08% (**b**), 20.07% (**c**), and 23.86% (**d**) (d.b.).

In general, dielectric constants and loss factors of walnut kernels and shells at seven temperatures and four respective MCs decreased with raised frequency from 10 to 3000 MHz (Figure 1, Figure 2, Figure 3 and Figure 4). For kernels with a MC of 4.21% (d.b.), the dielectric constants were smaller than 4.0 while the loss factors were less than 1.2 at seven temperatures. The extremely small dielectric properties resulted mainly from low ionic conductivity and water relaxation caused by low MC [36]. In addition, both dielectric constants and loss factors increased slightly and reached a local maximum at the frequency of about 1600 MHz. The phenomenon was caused by the joint effects of ionic conductivity and free water relaxation whose contribution to dielectric losses reached its maximum at about 2000 MHz as the frequency increased from 10 to 3000 MHz [36]. Liu et al. (2020) and Li et al. (2017) found similar trends in the dielectric properties of honey [37] and almonds [32], respectively, and the different frequencies corresponding to the local maximum were due to the different chemical composition of various foods. In comparison, dielectric properties of shells with the lowest MC (12.51% d.b.) decreased steadily with no local maximum as the frequency increased. It was because the contribution of free water relaxation to the dielectric loss was much smaller than that of ionic conductivity at relatively high MCs. Particularly, standard deviations of dielectric properties of walnut kernels with an MC of 4.21% (d.b.) were relatively larger than those of kernels with larger MCs owing to the greater effect of systematic errors on experimental results.

For kernels with MCs of 8.08%, 12.08%, and 16.23% (d.b.), and shells with MCs of 16.08%, 20.07%, and 23.86% (d.b.), their dielectric constants and loss factors decreased with increasing frequency with greater rates at lower frequencies (Figure 3 and Figure 4). For example, as the frequency rose from 10 to 300 MHz, dielectric constant and loss factor of kernels with a MC of 16.23% (d.b.) descended from 25.41 and 42.18 to 11.55 and 3.87, respectively, at a temperature of 20 °C (Figure 1d and Figure 2d), leading to decreasing amplitudes of 54.55% and 90.83%. However, as the frequency continued to increase from 300 to 3000 MHz, the dielectric constant and loss factor declined to 8.35 and 3.18, respectively, resulting in decreasing amplitudes of only 27.71% and 17.83%. The results were consistent with the theory in Feng et al. (2002) [36] and Gezahegn et al. (2021) [38]. To be specific, at frequencies between 10 and 300 MHz, the ionic conductivity dominated the dielectric dispersion and its contribution to DPs decreased rapidly with increasing frequency. As the frequency rose from 300 to 3000 MHz, the water relaxation was the dominant mechanism and varied slightly with increasing frequency. Those trends in dielectric properties were similar to those of tuna [18], edible fungi powder [39], and Camellia oleifera seed kernels [40].

### 3.3. Moisture and Temperature-Dependent Dielectric Properties

**Figure 5:**
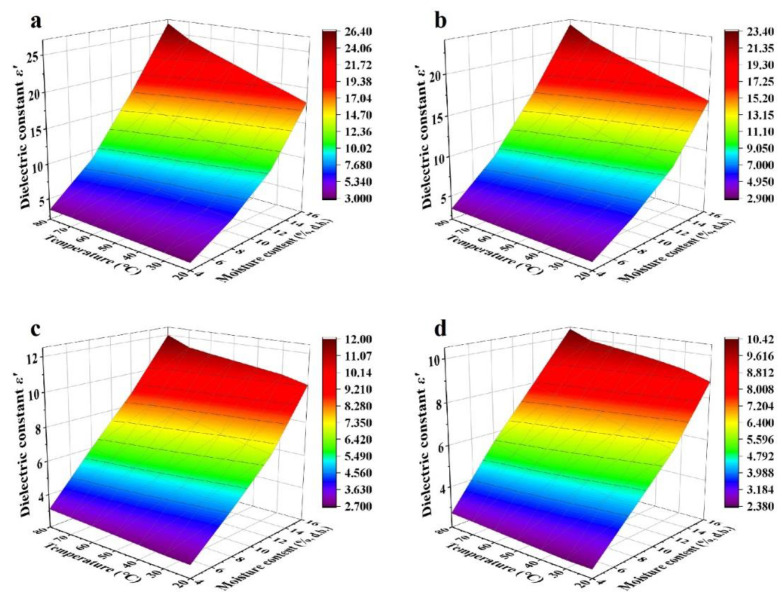
Moisture content and temperature-dependent dielectric constant (*ε′*) of walnut kernels at frequencies of 27 (**a**), 40 (**b**), 915 (**c**), and 2450 (**d**) MHz.

**Figure 6:**
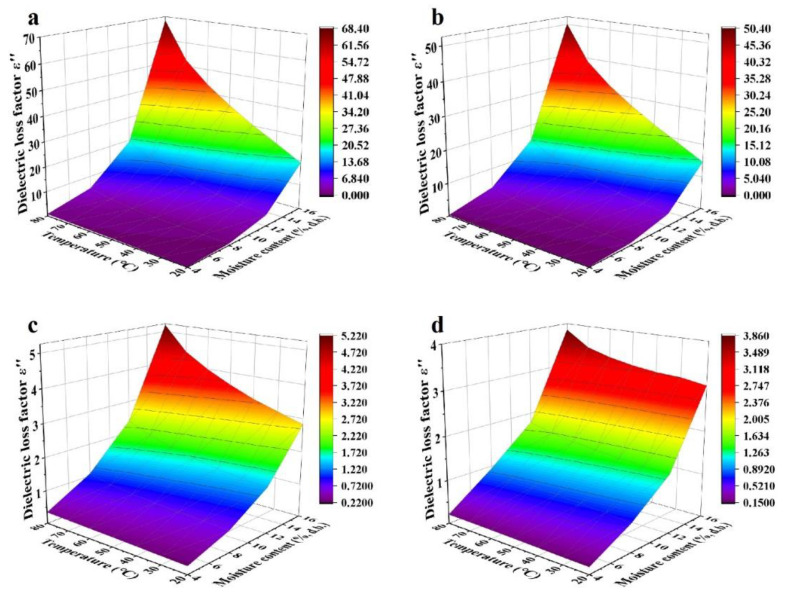
Moisture content and temperature-dependent dielectric loss factor (*ε″*) of walnut kernels at frequencies of 27 (**a**), 40 (**b**), 915 (**c**), and 2450 (**d**) MHz.

**Figure 7:**
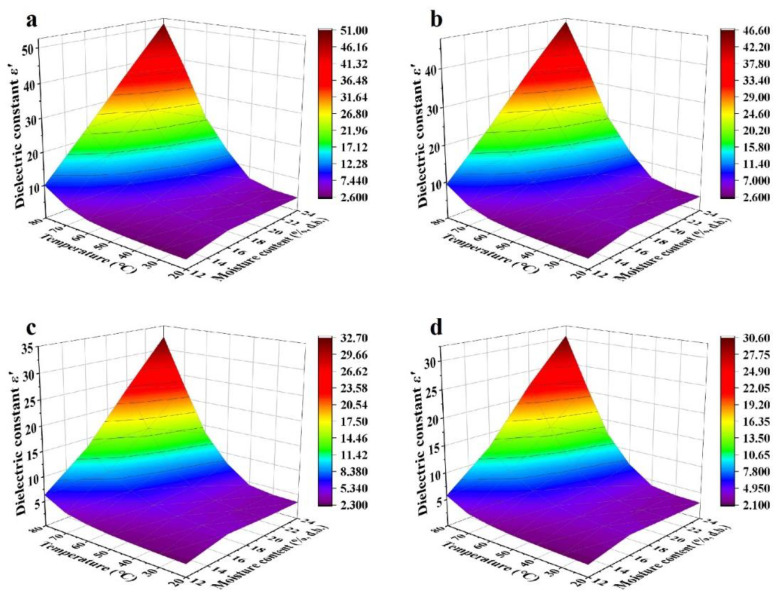
Moisture content and temperature-dependent dielectric constant (*ε′*) of walnut shells at frequencies of 27 (**a**), 40 (**b**), 915 (**c**), and 2450 (**d**) MHz.

**Figure 8:**
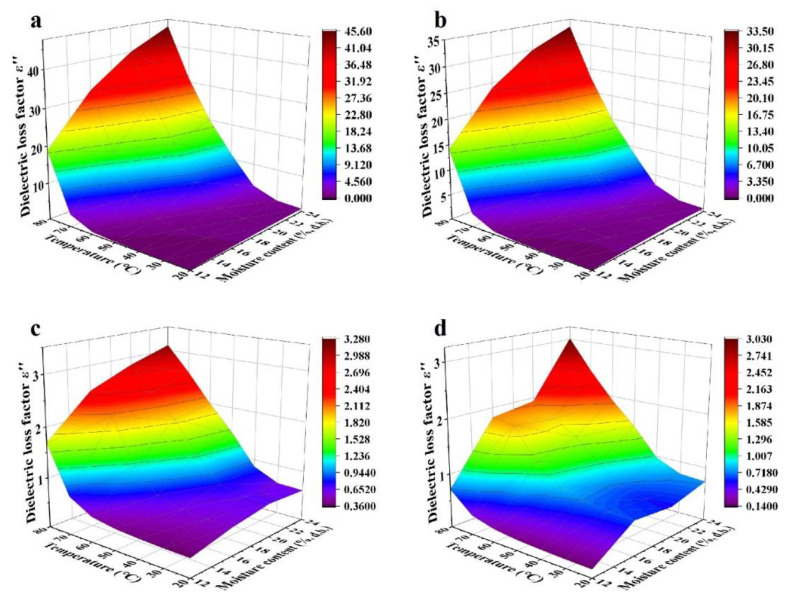
Moisture content and temperature-dependent dielectric loss factor (*ε″*) of walnut shells at frequencies of 27 (**a**), 40 (**b**), 915 (**c**), and 2450 (**d**) MHz.

Effects of temperature (20–80 °C) and MC (4.21%–16.23% d.b. for kernels and 12.51%–23.86% d.b. for shells) on dielectric properties of walnut components at frequencies of 27, 40, 915, and 2450 MHz are shown in Figure 5, Figure 6, Figure 7 and Figure 8. Generally, both dielectric constant and loss factor of walnut kernels and shells increased with increasing temperature and MC at a selected frequency. For instance, at a certain frequency of 27 MHz, dielectric constant *ε′* and dielectric loss factor *ε″* rose from 10.03 and 18.83 to 50.87 and 45.41, respectively, with raised MC from 12.51% to 23.86% (d.b.) at a fixed temperature of 80 °C; when the MC was fixed at 23.86%, *ε′* and *ε″* increased from 4.13 and 0.80 to 50.87 and 45.41, respectively, when the temperature increased from 20 to 80 °C. On the one hand, the enhancement of ionic conductivity by greater MC was the main cause of the increase in dielectric properties. To be specific, most water molecules in materials with low MCs were usually arranged in a monolayer form and bonded tightly with other polar molecules by hydrogen bonds, leading to inferior ionic conductivities. With the increase in the MC, the arrangement of water molecules transformed from the monolayer to a multilayer form and air voids in walnut kernels and shells were filled gradually, raising the ionic solubility and the water dipole mobility simultaneously; thus, improving the ionic conductivity [41]. On the other hand, as the temperature was elevated, the viscosity of materials was reduced, and the ionic migration and dipolar rotation were accelerated. Consequently, the dielectric constant and loss factor of walnut kernels and shells were enhanced by these two mechanisms. The results were found to be similar to those reported by Cao et al. (2018) for surimi [42]. Based on the aforementioned theory, during RF or MW drying, samples with larger MCs had greater dielectric constants and loss factors, making them tend to be heated rapidly, then the resulted high temperature leading to larger drying rates of samples. This phenomenon was commonly named as the “moisture levelling effect”, which was conducive to improving the uniformity of moisture distribution in products and the product quality stability during storage. Moreover, the increasing rates of dielectric constant and loss factor with MC were greater at higher temperatures, which might be caused by the synergistic effect of temperature and MC and are also reported by Li et al. (2017) and Jiang et al. (2020) in the research on dielectric properties of almonds [32] and *Agaricus bisporus* slices [43]. The phenomenon suggested that applying relatively low temperature at the initial RF or MW pasteurization stage helped to avoid the “heating runaway” effect.

Figure 5, Figure 6, Figure 7 and Figure 8 also show a trend that the variations in dielectric properties of kernels with MC from 4.21% to 16.23% (d.b.) were greater than those with temperatures from 20 to 80 °C whereas temperature had greater influence on dielectric properties of shells. For example, dielectric constant of walnut kernels at 20 and 80 °C dropped from 17.94 and 26.39 to 3.00 and 3.41, respectively, as the MC decreased from 16.23% to 4.21% (d.b.) under a frequency of 27 MHz, holding great decreasing amplitudes of 83.28% and 87.08%. By comparison, the *ε′* under MCs of 4.21% and 16.23% (d.b.) declined from 3.41 and 26.39 to 3.00 and 17.94 as the temperature decreased from 80 to 20 °C with decreasing amplitudes of only 12.02% and 32.02%. Yu et al. (2015) reported that dielectric properties of bulk canola seeds varied with temperature and MC following a similar trend [44]. Furthermore, Figure 5 and Figure 7 demonstrate that dielectric loss factors of kernels were larger than those of shells with corresponding MCs at the same frequency and temperature, resulting in greater heating rates for kernels as compared with those of shells.

### 3.4. Regression Models for Dielectric Properties

The established regression models for dielectric properties of walnut components as a function of temperature and MC at four frequencies of 27, 40, 915, and 2450 MHz are listed in Table 2. At frequencies in RF bands of 27 and 40 MHz, quadratic polynomial equations could predict moisture and temperature-dependent dielectric constant *ε′* of kernels and dielectric loss factor *ε″* of shells with the best fit, while cubic polynomial models were the best fit for *ε′* of shells and *ε″* of kernels. For frequencies of 915 and 2450 MHz in the MW band, cubic polynomial relationships were the best fit for dielectric properties of both walnut kernels and shells except for the *ε″* of shells at 915 MHz whose optimal fitting model was a quadratic polynomial equation. According to results of analysis of variance (ANOVA), all established models could predict the measured dielectric properties of walnut components with a good fit at a significance level of 0.0001 (*p* < 0.0001) and their coefficients of determination (*R*^2^) were all above 0.966. These results suggested that the developed mathematical models could predict the dielectric constants and loss factors of walnut kernels and shells with an adequate precision within a temperature range of 20–80 °C at MCs from 4.21% to 16.23% (d.b.) for kernels and 12.51% to 23.86% (d.b.) for shells and provide necessary data for further computer simulation.

### 3.5. Penetration Depth

The penetration depths of electromagnetic waves into walnut kernels and shells calculated based on the measured dielectric properties at four frequencies, four temperatures, and different MCs are listed in Table 3 and Table 4. The penetration depth was found to be smaller at greater frequencies, temperatures, and MCs. For example, the penetration depth *d_p_* of the electromagnetic wave into walnut kernels with a MC of 4.21% (d.b.) decreased from 1845.75 to 421.55 cm as the temperature increased from 20 to 80 °C at a frequency of 27 MHz. The *d_p_* dropped from 931.10 to 31.10 cm as the MC increased from 4.21% to 16.23% (d.b.) for kernels with a temperature of 40 °C at 27 MHz. When the frequency increased from 27 to 2450 MHz, the corresponding penetration depth in kernels with a MC of 8.08% under 20 °C declined from 656.77 to 6.29 cm. It could be concluded that the penetration depth of the RF wave was much larger than that of the MW wave, enabling the RF technology to be more suitable for heating walnut samples with large volumes because of the better heating uniformity. The penetration depths in shells were greater than those in kernels with corresponding MCs at the same frequency and temperature, making the electromagnetic waves penetrate through shells and interact with kernels directly. Research for *Agaricus bisporus* slices [43] and almonds [32] also revealed similar results that penetration depth increased with reduced frequency, temperature, and MC.

### 3.6. Moisture and Temperature-Dependent Thermal Properties

**Figure 9:**
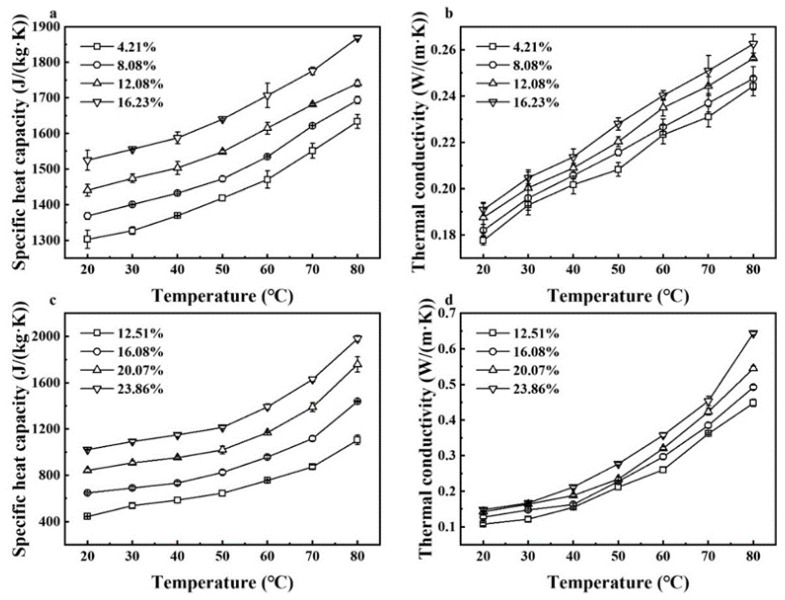
Moisture content and temperature-dependent specific heat capacity and thermal conductivity of walnut kernels (**a**,**b**) and shells (**c**,**d**).

Specific heat capacity and thermal conductivity of walnut kernels and shells all increased with raised temperature and MC (Figure 9). Taking walnut shells as an example (Figure 9c,d), their specific heat capacity and thermal conductivity increased from 444.50 J/(kg·K) and 0.11 W/(m·K) to 1980.41 J/(kg·K) and 0.64 W/(m·K), respectively, with increased temperature from 20 to 80 °C and MC from 12.51% to 23.86% (d.b.). Oriola et al. (2021) and Aviara and Haque (2001) also found the similar trends in the thermal properties of Jack bean seeds [45] and sheanuts [46], respectively. Since the specific heat capacity *C_p_* of water is a relatively high value of 4200 J/(kg·K), the *C_p_* of materials was larger with higher MC. Moreover, the addition of moisture filled the voids in kernels and shells and increased the mobility of water, leading to the increase in the thermal conductivity. Since the thermal conductivity of shells was much larger than that of kernels for in-shell walnuts, addition of extra hot air is required to maintain sample temperatures during RF or MW pasteurization.

The polynomial regression models describing the variation in thermal properties of walnut components with temperature and MC were developed and listed in Table 5. A two-factor interaction model and quadratic polynomial equation were the best fit for thermal conductivity and specific heat capacity of walnut kernels, respectively, and the cubic polynomial relationship was the best fit for the thermal properties of shells. Coefficients of determination (*R*^2^) of established models were all greater than 0.995, demonstrating that those mathematical models provided a precise prediction for thermal properties of walnut components.

## 4. Conclusions

Dielectric and thermal properties of both walnut kernels and shells were affected by temperature and MC, and dielectric properties decreased with increasing frequency. RF or MW heating rates of walnut kernels could be greater than those of shells because of the higher dielectric loss factors at the same temperature and MC. Penetration depth decreased with raised frequency, temperature, and MC, and great penetration depths in both kernels and shells at RF frequencies made the RF heating more effective for pasteurizing bulk and thick materials as compared with the MW method. Because of the lower dielectric loss factor and greater thermal conductivity of walnut shells than those of kernels, it is advisable to combine RF or MW heating with assisted hot air to maintain the temperature of shells. Both quadratic and cubic polynomial regression models provided the best fit for dielectric and thermal properties of walnut components. Consequently, the knowledge of dielectric and thermal properties of both walnut kernels and shells is conducive to developing rapid and effective RF or MW pasteurization technologies for in-shell walnuts at laboratory, pilot, and industrial scales, and provided necessary data for the further computer simulation.

## Figures and Tables

**Table 1:** True densities (mean ± SD over three replicates) of walnut kernels and shells at different moisture contents.

Moisture Content (%, d.b.)		True Density (g/cm^3^)
Kernel	4.21	1.019 ± 0.006 a
	8.08	1.012 ± 0.004 a b
	12.08	1.007 ± 0.003 b
	16.23	1.003 ± 0.003 b
Shell	12.51	0.988 ± 0.023 c
	16.08	1.021 ± 0.015 b c
	20.07	1.045 ± 0.006 a b
	23.86	1.067 ± 0.007 a

Different lower-case letters indicate that means are significantly different at *p =* 0.05 among different moisture contents.

**Table 2:** Regression equations of dielectric properties of walnut components at the 4 representative frequencies as a function of moisture content (*M*, %) and temperature (*T*, °C).

Frequency (MHz)	Material	Dielectric Properties	*R* ^2^
27	Kernel	*ε′* = 4.24 − 8.78 × 10^−2^ *T* − 0.37 *M* + 1.14 × 10^−2^ *TM* + 4.68 × 10^−4^ *T*^2^ + 6.62 × 10^−2^ *M*^2^	0.999
		*ε″* = −34.17 + 1.10 *T* + 9.48 *M* − 0.15 *TM* − 1.47 × 10^−2^ *T*^2^ − 0.95 *M*^2^ + 6.58 × 10^−4^ *T*^2^ *M* + 7.20 × 10^−3^ *TM*^2^ + 7.51 × 10^−5^ *T*^3^ + 3.31 × 10^−2^ *M*^3^	0.997
	Shell	*ε’* = −46.15 + 0.61 *T* + 7.64 *M* − 7.18 × 10^−2^ *TM* − 1.20 × 10^−2^ *T*^2^ − 0.34 *M*^2^ + 1.08 × 10^−3^ *T*^2^ *M* + 6.95 × 10^−4^ *TM*^2^ + 1.64 × 10^−5^ *T*^3^ + 5.23 × 10^−3^ *M*^3^	0.996
		*ε″* = 21.36 − 1.70 *T* + 0.31 *M* + 4.65 × 10^−2^ *TM* + 1.38 × 10^−2^ *T*^2^ − 4.06 × 10^−2^ *M*^2^	0.974
40	Kernel	*ε′* = 3.52 − 7.32 × 10^−2^ *T* − 0.17 *M* + 9.37 × 10^−3^ *TM* + 3.98 × 10^−4^ *T*^2^ + 5.19 × 10^−2^ *M*^2^	0.999
		*ε″* = −23.27 + 0.75 *T* + 6.51 *M* − 0.10 *TM* − 1.03 × 10^−2^ *T*^2^ − 0.66 *M*^2^ + 4.63 × 10^−4^ *T*^2^ *M* + 4.95 × 10^−3^ *TM*^2^ + 5.31 × 10^−5^ *T*^3^ + 2.33 × 10^−2^ *M*^3^	0.998
	Shell	*ε’* = −43.54 + 0.72 *T* + 6.81 *M* − 7.53 × 10^−2^ *TM* − 1.29 × 10^−2^ *T*^2^ − 0.29 *M*^2^ + 1.03 × 10^−3^ *T*^2^ *M* + 7.79 × 10^−4^ *TM*^2^ + 2.35 × 10^−5^ *T*^3^ + 4.09 × 10^−3^ *M*^3^	0.996
		*ε″* = 12.60 − 1.23 *T* + 0.55 *M* + 3.39 × 10^−2^ *TM* + 1.01 × 10^−2^ *T*^2^ − 3.79 × 10^−2^ *M*^2^	0.974
915	Kernel	*ε’* = 0.19 + 7.10 × 10^−3^ *T* + 0.59 *M* + 7.12 × 10^−2^ *TM* − 6.19 × 10^−4^ *T*^2^ − 3.09 × 10^−2^ *M*^2^ + 1.00 × 10^−5^ *T*^2^ *M* − 3.12 × 10^−4^ *TM*^2^ + 3.93 × 10^−6^ *T*^3^ + 1.85 × 10^−3^ *M*^3^	1.000
		*ε″* = −1.39 + 4.78 × 10^−2^ *T* + 0.40 *M* − 5.72 × 10^−3^ *TM* − 6.89 × 10^−4^ *T*^2^ − 3.26 × 10^−2^ *M*^2^ + 3.68 × 10^−5^ *T*^2^ *M* + 2.44 × 10^−4^ *TM*^2^ + 3.36 × 10^−6^ *T*^3^ + 1.39 × 10^−3^ *M*^3^	0.999
	Shell	*ε’* = −61.30 + 1.27 *T* + 8.64 *M* − 9.72 × 10^−2^ *TM* − 1.73 × 10^−2^ *T*^2^ − 0.37 *M*^2^ + 8.86 × 10^−4^ *T*^2^ *M* + 1.28 × 10^−3^ *TM*^2^ + 5.06 × 10^−5^ *T*^3^ + 5.61 × 10^−3^ *M*^3^	0.997
		*ε″* = −0.96 − 7.15 × 10^−2^ *T* + 0.25 *M* + 2.30 × 10^−2^ *TM* + 6.37 × 10^−4^ *T*^2^ − 7.64 × 10^−3^ *M*^2^	0.967
2450	Kernel	*ε′* = 0.28 − 1.10 × 10^−2^ *T* + 0.51 *M* + 8.95 × 10^−3^ *TM* − 3.94 × 10^−4^ *T*^2^ − 2.43 × 10^−2^ *M*^2^ − 3.77 × 10^−6^ *T*^2^ *M* − 3.40 × 10^−4^ *TM*^2^ + 3.07 × 10^−6^ *T*^3^ + 1.38 × 10^−3^ *M*^3^	1.000
		*ε″* = −1.72 + 2.90 × 10^−2^ *T* + 0.55 *M* − 1.60 × 10^−3^ *TM* − 5.17 × 10^−4^ *T*^2^ − 5.33 × 10^−2^ *M*^2^ + 2.09 × 10^−5^ *T*^2^ *M* + 2.01 × 10^−5^ *TM*^2^ + 2.61 × 10^−6^ *T*^3^ + 2.29 × 10^−3^ *M*^3^	0.999
	Shell	*ε’* = −49.63 + 1.20 *T* + 6.66 *M* − 8.69 × 10^−2^ *TM* − 1.72 × 10^−2^ *T*^2^ − 0.27 *M*^2^ + 8.49 × 10^−4^ *T*^2^ *M* + 1.04 × 10^−3^ *TM*^2^ + 5.25 × 10^−5^ *T*^3^ + 3.89 × 10^−3^ *M*^3^	0.996
		*ε″* = −22.15 − 7.87 × 10^−2^ *T* + 3.98 *M* + 1.48 × 10^−3^ *TM* + 1.06 × 10^−3^ *T*^2^ − 0.22 *M*^2^ + 1.32 × 10^−5^ *T*^2^ *M* − 5.48 × 10^−6^ *TM*^2^ − 6.34 × 10^−6^ *T*^3^ + 3.94 × 10^−3^ *M*^3^	0.976

**Table 3:** Penetration depth (cm) of electromagnetic waves into walnut kernels at different frequencies, moisture contents, and temperatures.

Moisture Content (%, d.b.)	Temperature (°C)	Penetration Depth (cm)			
		27 MHz	40 MHz	915 MHz	2450 MHz
4.21	20	1845.75 ± 108.31	1239.87 ± 73.91	27.59 ± 0.25	25.19 ± 0.04
	40	973.10 ± 73.58	645.63 ± 25.26	22.83 ± 0.01	16.43 ± 0.02
	60	604.89 ± 10.65	427.63 ± 2.83	18.89 ± 0.02	11.62 ± 0.01
	80	421.55 ± 5.41	325.36 ± 2.97	17.29 ± 0.02	10.37 ± 0.01
8.08	20	656.77 ± 44.57	424.37 ± 4.06	17.75 ± 0.07	6.29 ± 0.03
	40	435.42 ± 7.87	315.57 ± 6.41	16.39 ± 0.03	5.69 ± 0.00
	60	243.21 ± 5.65	188.68 ± 3.42	14.81 ± 0.05	5.38 ± 0.01
	80	109.35 ± 1.19	89.58 ± 1.22	11.94 ± 0.06	4.87 ± 0.01
12.08	20	131.12 ± 0.88	104.60 ± 0.41	10.03 ± 0.01	3.63 ± 0.00
	40	89.63 ± 0.29	72.14 ± 0.44	9.24 ± 0.00	3.57 ± 0.00
	60	62.18 ± 0.14	49.97 ± 0.15	8.03 ± 0.01	3.43 ± 0.00
	80	42.10 ± 0.21	33.91 ± 0.19	6.57 ± 0.02	3.12 ± 0.01
16.23	20	42.43 ± 0.91	35.61 ± 0.65	5.95 ± 0.05	1.93 ± 0.02
	40	31.10 ± 0.04	25.52 ± 0.01	5.23 ± 0.00	1.92 ± 0.00
	60	24.34 ± 0.04	19.91 ± 0.02	4.44 ± 0.00	1.85 ± 0.00
	80	18.26 ± 0.09	14.91 ± 0.07	3.55 ± 0.01	1.66 ± 0.00

**Table 4:** Penetration depth (cm) of electromagnetic waves into walnut shells at different frequencies, moisture contents, and temperatures.

Moisture Content (%, d.b.)	Temperature (°C)	Penetration Depth (cm)			
		27 MHz	40 MHz	915 MHz	2450 MHz
12.51	20	2648.13 ± 306.53	2015.44 ± 135.34	78.14 ± 9.05	18.97 ± 0.11
	40	2506.65 ± 194.41	1592.42 ± 99.18	73.97 ± 5.74	17.28 ± 0.08
	60	533.90 ± 45.83	427.54 ± 31.26	15.75 ± 1.35	13.75 ± 0.16
	80	37.25 ± 1.51	30.73 ± 1.22	1.10 ± 0.04	6.60 ± 0.07
16.08	20	983.50 ± 72.43	596.91 ± 19.48	19.22 ± 0.08	5.93 ± 0.01
	40	695.05 ± 53.40	481.09 ± 24.31	18.43 ± 0.13	5.54 ± 0.04
	60	103.81 ± 8.46	83.35 ± 6.83	11.53 ± 0.44	4.11 ± 0.10
	80	31.12 ± 0.33	25.74 ± 0.27	7.65 ± 0.03	3.78 ± 0.01
20.07	20	551.03 ± 3.04	344.42 ± 6.08	15.03 ± 0.01	6.40 ± 0.01
	40	261.02 ± 11.70	190.50 ± 10.86	13.96 ± 0.13	5.77 ± 0.05
	60	47.26 ± 0.88	38.38 ± 0.69	8.33 ± 0.05	4.00 ± 0.02
	80	29.92 ± 0.11	25.22 ± 0.09	8.86 ± 0.04	4.76 ± 0.01
23.86	20	453.58 ± 19.64	372.23 ± 5.47	15.87 ± 0.01	4.66 ± 0.00
	40	124.29 ± 7.13	101.60 ± 5.68	12.73 ± 0.27	3.99 ± 0.06
	60	45.68 ± 0.21	38.30 ± 0.22	9.57 ± 0.03	3.54 ± 0.01
	80	30.05 ± 0.27	25.75 ± 0.27	9.61 ± 0.09	3.57 ± 0.01

**Table 5:** Regression equations of thermal properties of walnut components as a function of moisture content (*M*, %) and temperature (*T*, °C).

Material	Thermal Properties		*R* ^2^
Kernel	Thermal conductivity	*k* = 0.16 + 0.001 *T* + 7.69 × 10^−4^ *M* + 1.16 × 10^−5^ *TM*	0.996
	Specific heat capacity	*C_p_* = 1237.31 − 0.26 *T* + 10.59 *M* + 5.90 × 10^−3^ *TM* + 5.63 × 10^−2^ *T*^2^ + 0.38 *M*^2^	0.997
Shell	Thermal conductivity	*k* = −0.41 + 8.77 × 10^−3^ *T* + 6.73 × 10^−2^ *M* − 8.54 × 10^−4^ *TM* − 4.54 × 10^−5^ *T*^2^ − 2.68 × 10^−3^ *M*^2^ + 4.83 × 10^−6^ *T*^2^ *M* + 1.52 × 10^−5^ *TM*^2^ + 4.03 × 10^−7^ *T*^3^ + 3.79 × 10^−5^ *M*^3^	0.996
	Specific heat capacity	*C_p_* = −216.30 + 31.28 *T* − 4.50 *M* − 0.11 *TM* − 0.73 *T*^2^ + 2.97 *M*^2^ + 1.45 × 10^−2^ *T*^2^ *M* − 2.50 × 10^−2^ *TM*^2^ + 4.77 × 10^−3^ *T*^3^ − 3.64 × 10^−2^ *M*^3^	0.998

## Data Availability

The datasets generated for this study are available on request to the corresponding author.
